# Periodontal Plastic Microsurgery in the Treatment of Deep Gingival Recession after Orthodontic Movement

**DOI:** 10.1155/2013/851413

**Published:** 2013-11-07

**Authors:** Sérgio Kahn, Walmir Júnio de Pinho Reis Rodrigues, Marcos de Oliveira Barceleiro

**Affiliations:** ^1^Department of Periodontics, Veiga de Almeida University (UVA), Rua Ibituruna 108, Tijuca, 20271-021 Rio de Janeiro, RJ, Brazil; ^2^School of Dentistry, Serra dos Órgãos University Center (UNIFESO), Avenida Alberto Torres 111, Alto, 25964-004 Teresópolis, RJ, Brazil; ^3^Department of Dentistry, School of Dentistry, Fluminense Federal University (UFF), Rua Silvio Henrique Braune 22, Centro, 28625-650 Nova Friburgo, RJ, Brazil

## Abstract

Gingival recession is a condition that affects a large portion of the young and adult population and negatively affects the aesthetic aspects of the smile. Many factors are related to its development, including orthodontic movement beyond the osseous limits. Many treatment options have been proposed to cover the exposed root surface. The aim of this article was to describe three cases where a subepithelial connective tissue graft was performed, using a microsurgical technique, in the treatment of deep gingival recession after orthodontic treatment. This technique resulted in successful root coverage and keratinized tissue gain, improving the gingival esthetic pattern.

## 1. Introduction

Gingival recession can be defined as the location of marginal periodontal tissues apical to the cementoenamel junction [1]. This condition is prevalent in the young and adult populations [2]. The prevalence, extension, and severity of gingival recession have been observed to increase with age, affecting 79% of adults >40 years old in a representative sample of the Brazilian population [3]. It occurs in patients with either good or poor oral hygiene [4] and has multiple etiological factors [5] like traumatic tooth brushing [6], dehiscence, smoking, biological width invasion, inflammation, occlusal trauma, piercings [7], and orthodontic movement.

Although controversial, the scientific evidence demonstrates that gingival recession can develop in patients who undergo orthodontic movement [8, 9]. Furthermore, some studies have confirmed a positive correlation between the increase of severity and extension of gingival recession and orthodontic treatment [10].

The main factors related to the occurrence of gingival recession related to orthodontic movement are tooth movement beyond the osseous limits of the alveolar process and excessive tooth proclination during treatment, especially in adult patients [11, 12].

Thin gingival biotypes and the gingival inflammation associated with biofilm were observed as the risk factors for the development of gingival recession associated with orthodontic treatment [13].

There are several options for the treatment of gingival recession, including the free gingival graft [14], the sliding flap [15], the double pedicle flap [16], the subepithelial connective tissue graft [17], the enamel matrix derivative, the acellular dermal matrix, and growth factors [18–20].

The scientific evidence shows that the subepithelial connective tissue graft promotes higher levels of root coverage, high predictability and provides more gingival thickness [18–20].

As new techniques and materials are developed, new surgical approaches are necessary to minimize the surgical trauma and overcome the limitations related to the manual ability and natural vision of the surgeons. The incorporation of a surgical microscope to periodontal plastic surgery provides better illumination and adequate magnification to increase the precision of a surgeon's surgical skill. Thus, minimally invasive techniques were developed to minimize tissue trauma and allow primary wound closure [21–23].

The aim of this study is to report three cases where a subepithelial connective tissue graft was performed using a microsurgical approach to treat deep gingival recession after orthodontic treatment.

## 2. Case Reports

Three patients from a private dental clinic with aesthetic complaints related to deep gingival recession were included in this report. All patients were healthy and nonsmokers and signed an informed consent. The details about each patient are described as follows.

**Figure 1:**
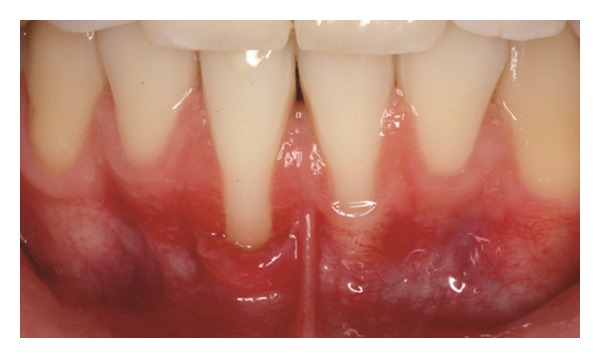
Initial aspect of patient 1 showing Miller Class III localized gingival recessions at the mandibular central incisors.

Patient 1: 27-year-old white female, having previous orthodontic treatment over a period of 5 years, presenting with a thin gingival biotype [24] and Miller [25] Class III gingival recession at the mandibular central incisors (Figure 1).

Patient 2: 26-year-old white female, who started orthodontic treatment approximately 3 years earlier, presenting with a thick gingival biotype [24] and Miller [25] Class III gingival recession at the left mandibular central incisor (Figure 10).

Patient 3: 26-year-old white male, who started orthodontic treatment approximately 2 years earlier, presenting with a thin gingival biotype [24] and Miller [25] Class II gingival recession at the right mandibular central incisor (Figure 17).

### 2.1. Initial Therapy

All patients were submitted to a plaque control program, which included oral hygiene instructions, scaling and root planning using an ultrasonic device, and crown polishing.

### 2.2. Surgical Therapy

All surgical procedures were performed by a single operator. A single 8 mg dose of dexamethasone was given to each patient one hour before surgery. Extraoral antisepsis was performed with a degerming agent and intraoral antisepsis was performed with a 0.12% chlorhexidine rinse. Mepivacaine (2.0%) with 1 : 100.000 epinephrine was used as an anesthetic solution.

All cases were performed using the microsurgical approach for root coverage, as described by De Campos et al. [22]. Initially, the exposed root surface was planned with a McCall 13/14 curette, followed by finishing burs. Citric acid gel (pH 1) was applied for 3 minutes. The surface was then washed for 45 seconds with a physiologic solution.

**Figure 2:**
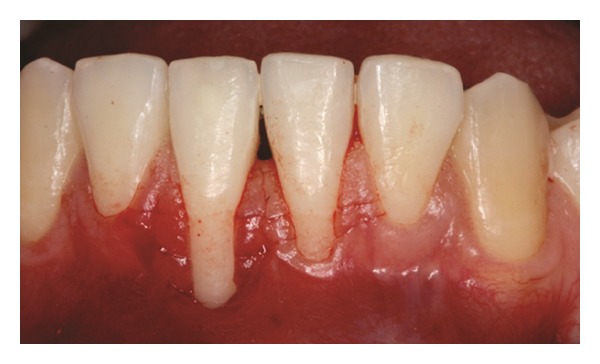
First and second incisions.

Both papillae adjacent to the recession were incised at buccal aspect. The first papillary incisions were horizontal at the level of the cementoenamel junction. The second papillary incisions were oblique and made at an apical level from those previously performed (Figures 2, 11 and 18). A third intrasulcular incision was performed to connect the papillary incisions. A partial thickness flap was performed from the second incision, allowing the removal of the gingival epithelium between the two incisions using Castroviejo scissors.

**Figure 3:**
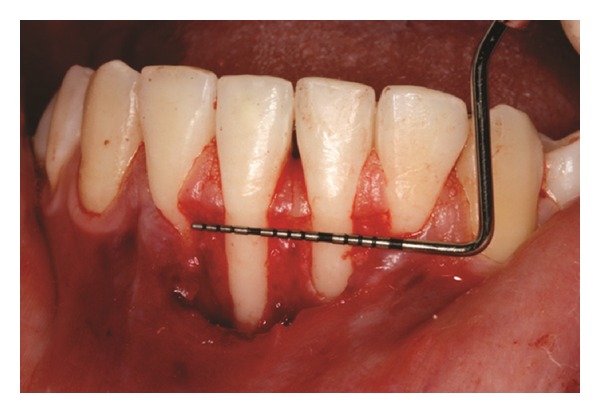
Measurement of recipient site after performing a partial thickness flap.

**Figure 4:**
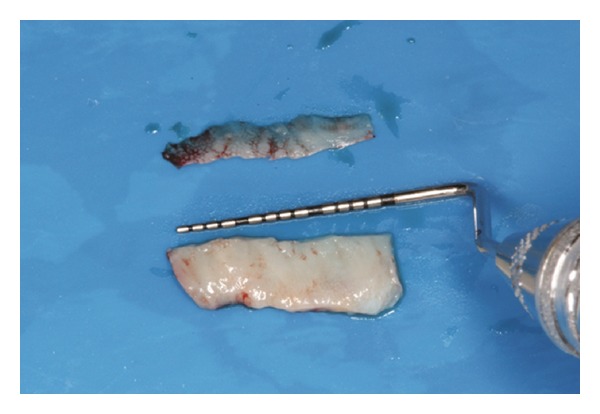
Connective tissue graft after the removal of the epithelium.

**Figure 5:**
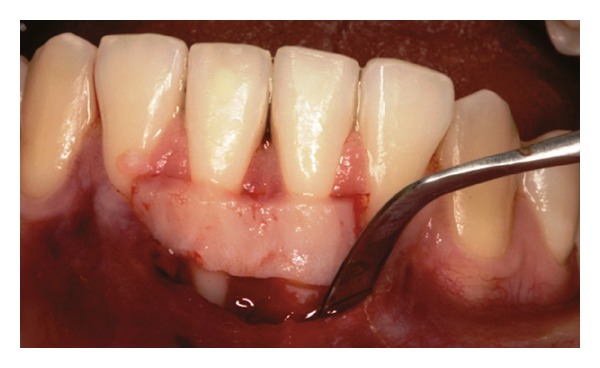
Graft stabilization at recipient site.

**Figure 6:**
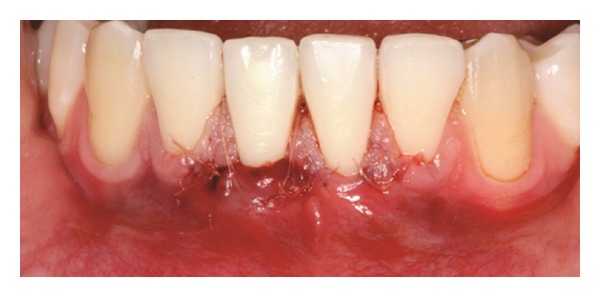
Microsutures.

The recipient site was measured (Figure 3) using a periodontal probe and these measurements were transferred to the donor site. A palatal connective tissue graft was harvested from the area distal to the canine and anterior to the mesial aspect of the first molar on the same side of the surgery. The epithelium at the graft was removed (Figures 4 and 12). After stabilizing the graft in the correct position (Figures 5, 13, and 19) using 6-0 vicryl sutures, the microsutures for flap approximation and coaptation were performed using 6-0 and 8-0 vicryl sutures, respectively (Figures 6, 14, and 20). The palate was then sutured with a continuous basting suture using a 4-0 silk thread. A thin layer of periodontal dressing was applied at both surgical sites.

### 2.3. Postoperative Care

**Figure 7:**
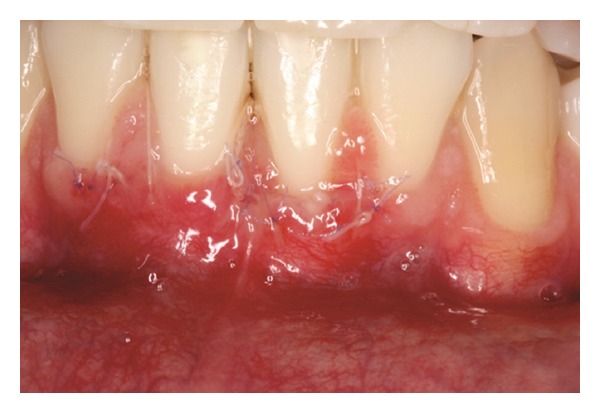
Seven-day follow-up of recipient site.

**Figure 8:**
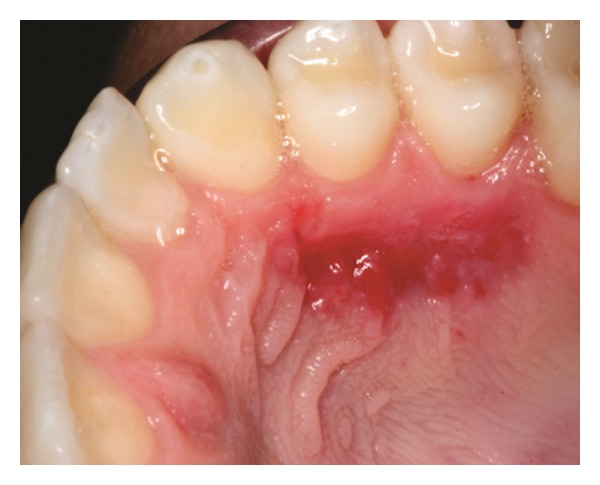
Seven-day follow-up of donor site.

**Figure 9:**
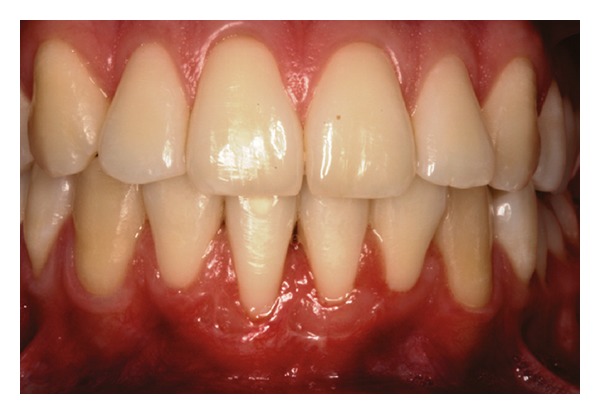
Thirty-day follow-up.

**Figure 10:**
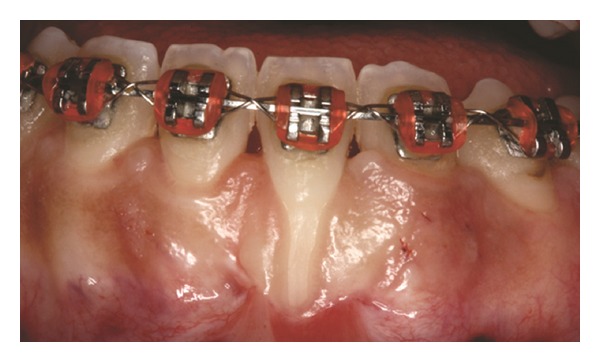
Initial aspect of patient 2 showing Miller class III localized gingival recession at the mandibular central left incisor.

**Figure 11:**
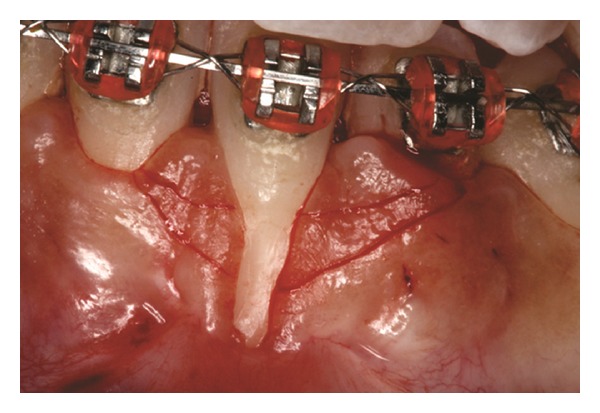
First and second incisions.

**Figure 12:**
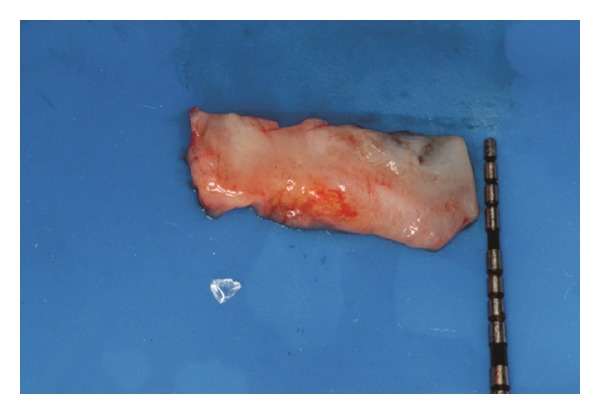
Connective tissue graft after the removal of the epithelium.

**Figure 13:**
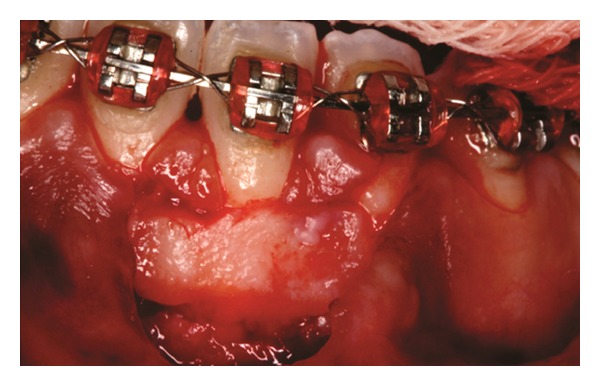
Graft stabilization at recipient site.

**Figure 14:**
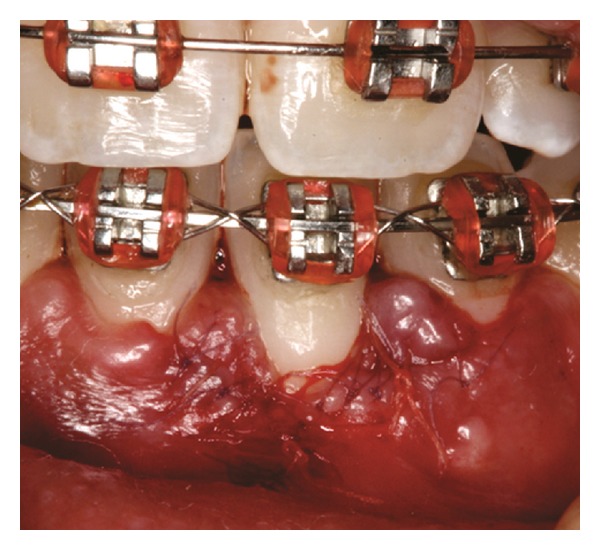
Microsutures.

**Figure 15:**
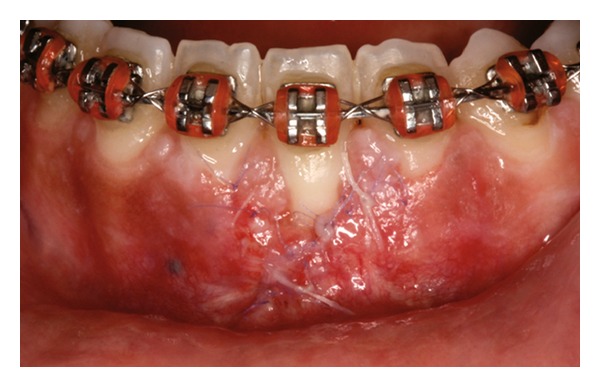
Seven-day follow-up of recipient site.

**Figure 16:**
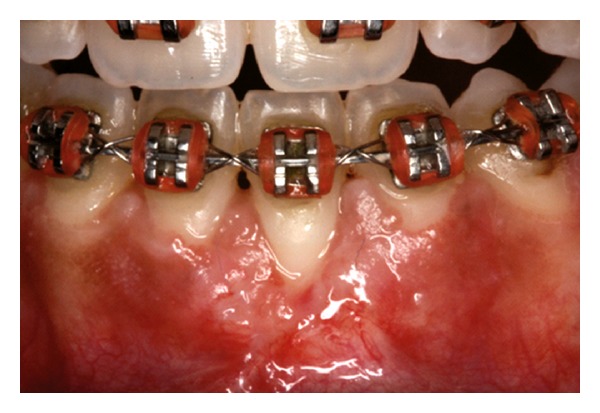
Thirty-day follow-up.

**Figure 17:**
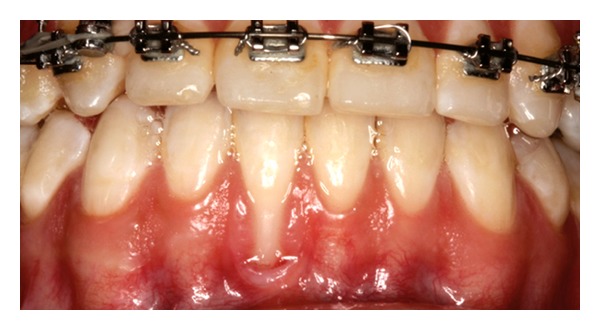
Initial aspect of patient 3 showing Miller Class II localized gingival recession at the mandibular central right incisor.

**Figure 18:**
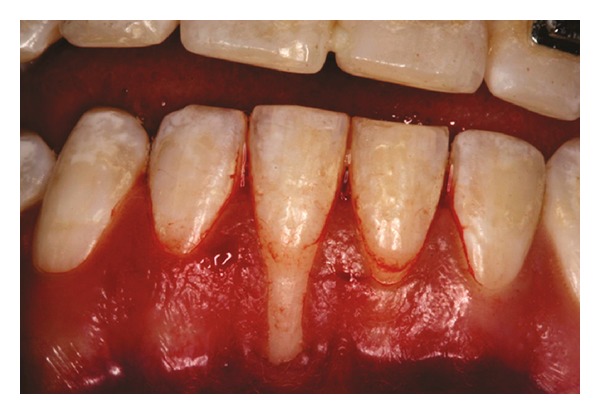
First and second incisions.

**Figure 19:**
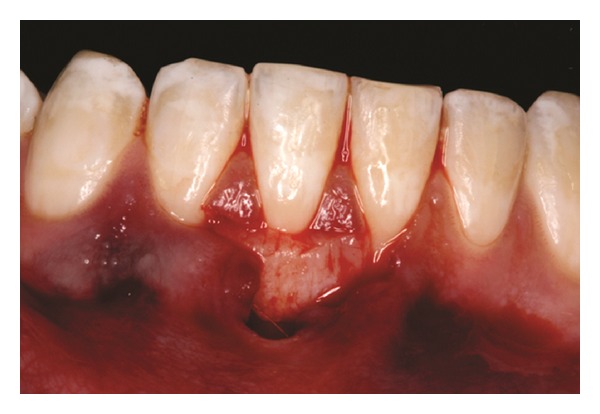
Graft stabilization at recipient site.

**Figure 20:**
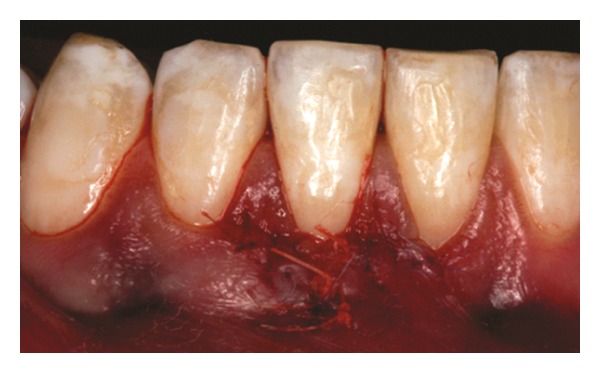
Microsutures.

**Figure 21:**
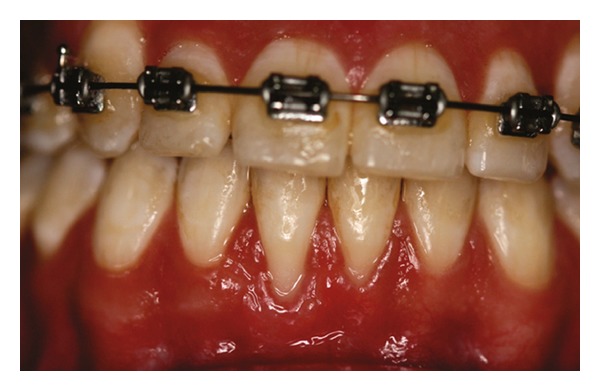
Follow-up visit. Patient 3 presented incomplete root coverage.

**Figure 22:**
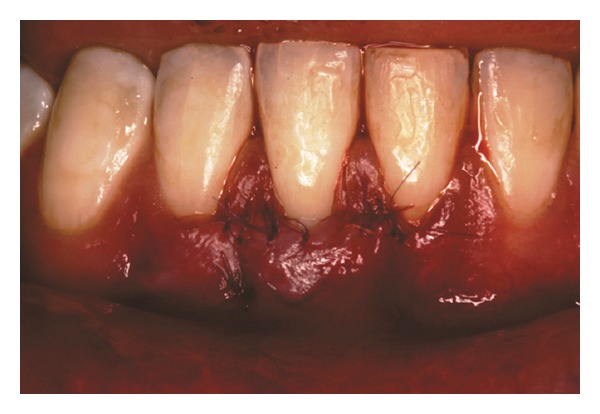
Second surgical procedure to achieve complete root coverage.

**Figure 23:**
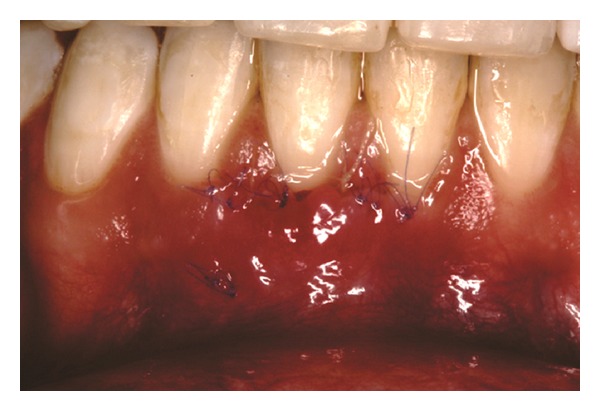
Seven-day follow-up of recipient site.

**Figure 24:**
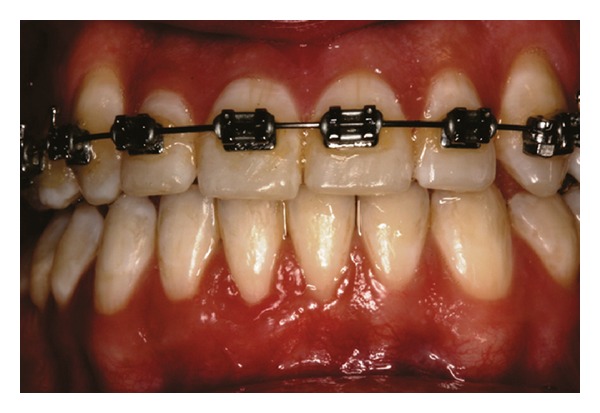
Thirty-day follow-up after the second intervention.

The patients were instructed to use Amoxicillin 500 mg t.i.d. for 7 days, Dexamethasone 4 mg b.i.d. in the first 24 hours and sodium dipyrone 1 g q.i.d. in the first 48 h. Furthermore, tooth brushing was discontinued around the surgical sites for three weeks and plaque control was provided by rinsing with a 0.12% chlorhexidine solution twice a day. After this period, the patients were instructed to use a unituft brush to clean the site. After 7 days, the periodontal dressing and sutures were removed (Figures 7, 8, and 15) and the patients were called for follow-up visits (Figures 9 and 16). During the follow-up visits, Patient 3 presented with incomplete root coverage after the initial surgical procedure. A second surgical procedure was performed to provide complete root coverage (Figures 21, 22, 23, and 24).

## 3. Discussion

Orthodontic movement is one of the important factors in the development of gingival recession. However, the scientific evidence related to this topic is scarce and controversial, since many studies use a limited sample and lack well-established evaluation criteria [11].

The results of several studies have demonstrated a higher prevalence and severity of gingival recession after orthodontic treatment. Nevertheless, some authors have observed that gingival recession related to orthodontic treatment affects a small portion of patients, and although there was an increase in severity, it did not result in severe clinical consequences [8, 10, 13].

It was observed that the proclination of incisors is related to the development of recession [11, 12]. However, Vasconcelos et al. [9] found that retroclination was also related to an increase in the severity of gingival recession. On the other hand, Djeu et al. [26] did not find a correlation between the proclination of mandibular central incisors and gingival recession.

With regards to gingival thickness, Yared et al. [12] observed that free gingival margins with a thickness <0.5 mm presented greater and more severe recession associated with mandibular central incisors. However, one study using a human sample found that the mean amount of initial keratinized gingiva did not predispose the mandibular incisors and canines to gingival recession [27]. An animal study found that central incisor sites submitted to gingival grafts showed less gingival recession and maintenance of gingival thickness after vestibular orthodontic movement [28].

Kao and Pasquinelli [24] classified the periodontal biotype as thin or thick. Thin biotypes present a thin underlying bone, characterized by bony dehiscence and fenestration, which reacts to insults and disease with gingival recession. The presence of a thin biotype was identified as a possible predictor of gingival recession in a study by Melsen and Allais [13]. The present study reported two cases, 1 and 3, which had thin biotypes, since a highly scalloped soft tissue and bony architecture and a minimal amount of attached gingiva were observed.

The surgical manipulation of thin gingival biotypes imposes some challenges for the surgeons, since there is a higher possibility of flap dilacerations and/or perforations, which can interfere in the final result of the surgical treatment. Thus, it is possible that the incorporation of microsurgical techniques using appropriate illumination and magnification for more precise flap elevation and sutures can lead to primary wound closure [21–23].

According to Miller's [25] classification, complete root coverage can be anticipated in Classes I and II, partial root coverage is expected in Class III and root coverage is not anticipated in Class IV. The presented cases were classified as Class II or III, and satisfactory root coverage was achieved in all cases. Patient 3 had a Miller Class II recession and was submitted to a second surgical procedure, since complete root coverage was not achieved with the first procedure and the gingival margin remained disharmonious. The reasons for this fact can be related to the initial recession depth as well as to the quality of the soft and hard tissue [18].

Within the limits of this study, a subepithelial connective tissue graft using a microsurgical approach resulted in successful root coverage and increased keratinized tissue, improving the gingival esthetic pattern in cases where deep gingival recession was associated with orthodontic movement.
